# Quality testing of veterinary antimicrobial products used for livestock in Vietnam, 2018–2019

**DOI:** 10.1371/journal.pone.0247337

**Published:** 2021-03-03

**Authors:** Huong Luu Quynh, Thuy Nguyen Thi Bich, Long Ta Hoang, Vera Irene Erickson, Pawin Padungtod

**Affiliations:** 1 National Institute of Veterinary Research, Hanoi, Vietnam; 2 Department of Animal Health, National Center for Veterinary Drugs Testing No. 1, Hanoi, Vietnam; 3 Food and Agriculture Organization of the United Nations, Vietnam Country Office, Hanoi, Vietnam; Nitte University, INDIA

## Abstract

Access to quality veterinary antimicrobial products contributes to efficient treatment of diseases in Vietnamese livestock and to reducing antimicrobial resistance (AMR). Poor quality antimicrobial drugs can lead to treatment failure, potentially influencing the inappropriate use of antimicrobials products, including increasing the dose, combining drugs, or changing to a broader spectrum antimicrobial. The objective of the study was to determine the actual concentration of antimicrobial active ingredient (AAI) in commercially available veterinary antimicrobial products as an indicator of their quality. A total of 144 veterinary antimicrobial products were purchased from randomly selected veterinary drug stores in 34 districts in eight provinces. For the qualitative analysis, we observed criteria linked to form, colour, and labelling information according to the Department of Animal Health regulations. For the quantitative analysis, high-performance liquid chromatography was used to determine the actual concentration of AAI in each sample. Of the 144 samples, 131 (91%) met the national standard of quality of being within ±10% of the labelled concentration. Ten antimicrobials (6.9%) contained less than half of the labelled content concentrations. Veterinary antimicrobial product quality control is an important part of addressing AMR. To support the national action plan to lower AMR, a veterinary drug quality control program should be implemented at all stages of the supply chain to assure high quality drugs and effective treatment of sick animals.

## Introduction

High quality veterinary antimicrobial products are important for efficient treatment of diseases in livestock, aquaculture and companion animals. Access to high quality veterinary antimicrobial products is an important part of the effort to reduce antimicrobial resistance (AMR) in Vietnamese livestock, aquaculture and public health sector [1].

Treatment failure could be the result of using a poor-quality drug with a lower concentration of antimicrobial active ingredient (AAI), or a sub-therapeutic AAI concentration [1]. If the concentration of the AAI is lower than stated on the label, the pathogens could be exposed to sub-therapeutic levels of antimicrobials. Sub-therapeutic treatment might not kill all the bacteria, allowing bacteria resistant to the antimicrobial drugs to survive, thereby contributing to AMR in animals [2]. If a farmer experiences treatment failure, it is likely that a more aggressive antimicrobial type, or an antimicrobial listed as critically important for human treatment, could be used. If a high-quality antimicrobial product had been used as the first treatment option, that would have been sufficient [1].

Higher concentrations of AAI can also lead to undesired side effects and antimicrobial residues in animal derived products. Higher than labelled concentrations can lead to supratherapeutic concentrations in treated animals that can select bacteria able to survive and disseminate AMR [1]. If there was a higher concentration of AAI in the drug, and a farmer followed the drug manufacturer’s instruction on withdrawal time, there could be residue of AAI in the product.

In the Vietnamese Ministry of Agriculture and Rural Development (MARD), the National Center of Veterinary Medicine Control I and II, under the Department of Animal Health (DAH), is responsible for conducting tests and assessing veterinary drug quality [3]. In June 2016, MARD issued a circular stating quality requirements for all veterinary drugs sold in Vietnam. The concentration of AAI in a product is allowed to deviate within ±10% from the stated concentration on the product label [3].

A study in the Mekong Delta area in Vietnam, published in 2019, investigated the quality of antimicrobials used in the poultry production. Of 20 samples collected from poultry farms, 6 (30.8%) had AAI concentrations within 10% of the labelled content, 8 (39.6%) contained AAI concentrations higher than the labelled content, and 6 (29.7%) had AAI concentrations less than 90% of the content. In total, only 15% of the tested products in this study contained all antimicrobials within ±10% of the concentration stated on the label [4]. Another study investigated the quality of antimicrobials used in Vietnamese white leg shrimp production. The results from this study found that only 1 of 25 products contained AAI within ± 10% of the labelled content [5].

We conducted this study to provide evidenced based data to advocate for policy changes in addressing issues of AMR and to ensure access to quality veterinary antimicrobial products for effective treatment of diseases in livestock, aquaculture and companion animals. The objective of the study was to determine the actual concentration of AAI of three antimicrobial groups; tetracyclines, sulphonamides and β-lactams, as a quality indicator of commercially available veterinary drugs.

## Materials and methods

### Collection of antimicrobial products

Between September 2018 and April 2019, we collected veterinary antimicrobial products from districts in eight of the 63 provinces of Vietnam. We included Thai Nguyen, Vinh Phuc, Nam Dinh, Hai Phong, Binh Dinh, Dak Lak, Dong Nai and Dong Thap provinces. These were similar to other provinces where previous studies had been conducted on antimicrobial usage in farm animals. In each province, three districts were randomly chosen for sampling. From each district, one veterinary drug store with a business certificate was randomly chosen from list of all veterinary drug stores using random number selection. In each selected store we purchased products containing tetracycline, sulphonamide and beta-lactam, including injectable samples commonly indicated for use in pigs, and powder samples commonly used for chickens [6, 7]. The sample size of 72 products for either pigs or chickens was sufficient to detect at least one failed product if the prevalence of failure was 5% at 95% confidence level (n = 60) with additional 20% attrition (n = 12).

### Antimicrobial analysis

Samples were analysed by the National Institute of Veterinary Research (NIVR) in collaboration with the National Center for Veterinary Drug Testing No.1. The test parameters included qualitative and quantitative analysis. The qualitative analysis was a visual observation of the parameters of each product, including form (liquid or powder), colour, and labelling according to the DAH regulations. For the quantitative analysis, high-performance liquid chromatography (HPLC), a laboratory-based method often used to determine the quality of pharmaceutical drugs, was used to determine the actual concentration of AAI in the sample. The product passed the quantitative analysis if the concentration of the AAI was within ± 10% of the labelled concentration.

### Ethical issues

The national standard procedures and analysis/testing methods for quality testing of veterinary drugs was followed (QCVN 01–187:2018/BNNPTNT; TCVN I-2:2017) and were in accordance with the law [3]. All specialized laboratory equipment was available and experienced staff were trained to conduct the testing [2].

## Results

A total of 144 veterinary antimicrobial drug products were analysed with HPLC; 72 powered samples and 72 injectable samples. Eighty one out of 144 products (56%) contained more than one AAI. Therefore, in total, 227 analyses were conducted. All of the products (100%) passed the qualitative assessment of visual observation of the form (liquid or powder), colour, and labelling according to the DAH regulations. For the quantitative analysis, of the 227 antimicrobials tested, 210 (88%) passed the quantitative test [S1 Table].

Of the 144 products, 13 (9%) contained one or more AAI that deviating more than ±10% from the stated concentration and therefore did not pass the quantitative test. Of these, 6 (4%) were in powder form and 7 (5%) were injectables. Of the 227 AAI tested, 79 samples (35%) had higher AAI concentration than what was stated on the label (>100%). and 10 samples (4.4%) had an actual concentration lower than 50% from what was stated on the label. The median absolute deviation was 65% (IQR = 49%). One product claimed to contain florfenicol (10g/100ml) and doxycycline (10g/100ml), but only contained florfenicol within the accepted range and contained 0% of doxycycline [Table 1].

**Table 1:** Antimicrobial active ingredients, failed samples and the range of deviation of products tested by high-performance liquid chromatography to determine concentration of antimicrobial active ingredients.

Antimicrobial active ingredient	Number of failed samples	Number of tested samples	Range of deviation of failed samples (%)
Amoxicillin	1	35	120
Ampicillin	4	16	8–53
Colistin	0	21	
Diaveridin	0	4	
Doxycycline	2	21	0–70
Erythromycin	0	1	
Florfenicol	0	7	
Lincomycin	0	1	
Oxytetracycline	4	25	9–65
Spiramycin	0	1	
Streptomycin	0	2	
Sulfachloropyrazine / Sulfaclozine	2	4	32–132
Sulfadiazine	0	3	
Sulfadimethoxine	0	5	
Sulfadimidine / Sulfamethazine	0	11	
Sulfaguanidine	0	2	
Sulfamethoxazole	0	18	
Sulfamethoxypyridazine	1	4	23
Sulfaquinoxaline	0	1	
Tetracycline	0	1	
Thiamphenicol	1	4	37
Tiamulin	0	1	
Trimethoprim	2	26	31–118
Tylosin	0	12	

## Discussion

This study indicated that approximately one in eight (12%) of veterinary antimicrobial products purchased in the sampled drug stores did not meet the national quality standard of an actual concentration of AAI within ± 10% deviation of what is stated on the label. Other studies on antimicrobial drug quality in Vietnam found 28.8% of antimicrobials for chickens deviated more than ± 10% from the labelled concentration [4]. In antimicrobial products used in aquaculture, two studies found 1/25 and 6/21 of the products had an actual concentration within ± 10% of the labelled concentration [5, 8]. Compared to previous studies, the rate of products analysed in this study with an actual concentration within ± 10% of the labelled concentration was higher.

Only a limited number of antimicrobial groups and antimicrobials were analysed in this study due to limited time and funding. It is possible that the analysed veterinary antimicrobial products contained other types of antibiotics or antimicrobials than the ones labelled and investigated in this study.

## Conclusions

When purchasing veterinary antimicrobial drug products from a veterinary drug store in Vietnam, there is a risk of buying a product of poor quality. Poor quality products, that expose livestock to sub-therapeutic antimicrobial levels that result in treatment failure, contribute to increasing levels of AMR. Similarly, AAI concentrations above the stated concentration can result in antimicrobial residues in animal derived products that result in hazards for human health and trade. Veterinary antimicrobial product quality control is an important part of addressing AMR. As drug quality can be compromised at any level, we recommend that quality control and assurance should involve all levels of the supply chain; from producer to distribution to storage in veterinary drug stores, as well as storage of drugs at user point. Correct label information is essential so that veterinary professionals and farmers can safely handle, administer and store the drugs. Labels should be examined to determine if all the necessary information, such as indications, withdrawal times, storage and expiry date, are clearly stated.

## Supporting information

S1 Table Qualitative and quantitative analysis of 144 veterinary antimicrobial product samples. (DOCX)
