# Supplementary material for: Quality testing of veterinary antimicrobial products used for livestock in Vietnam, 2018–2019
Source: PLoS One. 2021 Mar 3;16(3):e0247337. doi: 10.1371/journal.pone.0247337 (PMC7928525; doi:10.1371/journal.pone.0247337)
Supplement: S1 Table — (DOCX) [file pone.0247337.s001.docx]

# S1 Table: Qualitative and quantitative analysis of 144 veterinary antimicrobial product samples

| **No** | **Sample code** | **Antimicrobial active ingredient** | **Qualitative Observation of form, colour, and labelling** | **Quantity of antimicrobial active ingredient on the label** | **Actual quantity of antimicrobial active ingredient in the product** | **Method** | **Conclusion** |
| --- | --- | --- | --- | --- | --- | --- | --- |
|  | S1 | Florfenicol | Liquid, yellow. Bottle 100ml | 10g/100ml | 10.75  g/100ml | DP-AA-58 | Pass (107.5%) |
|  |  | Doxycycline hyclate |  | 10g/100ml | 10.12  g/100ml | DP-AA-48 | Pass (101.2%) |
|  | S2 | Florfenicol | Liquid, yellow. Bottle 100ml | 10g/100ml | 10.18  g/100ml | DP-AA-58 | Pass (101.7%) |
|  |  | Doxycycline hyclate |  | 10g/100ml | 0g/100ml | DP-AA-48 | Fail (0%) |
|  | S3 | Oxytetracycline | Liquid, tawny. Bottle 20ml | 200  mg/ 1ml | 188.6  mg/1ml | DP-AA-91 | Pass (94.28%) |
|  | S4 | Amoxicilline | Suspension, Barley white. Bottle 100ml | 14  g/100ml | 13.26  g/100ml | DP-AA-07 | Pass (94.7%) |
|  | S5 | Amoxicilline | Suspension, Barley white. Bottle 100ml | 15  g/100ml | 13.62  g/100ml | DP-AA-07 | Pass (90.79%) |
|  | S6 | Ampicillin | Powder, white. Bottle 500mg | 500  mg/500mg | 498.4  mg/500mg | DP-AA-05 | Pass (99.7%) |
|  | S7 | Sulfadimidine | Liquid, Egg yellow. Bottle 100ml | 200  mg/1ml | 203.2  mg/1ml | DP-AA-114 | Pass (101.6%) |
|  |  | Trimethoprim |  | 40  mg/100ml | 41.7  mg/100ml | DP-AA-131 | Pass (104.2%) |
|  | S8 | Tylosin | Liquid, transparent. Bottle 100ml | 500  mg/100ml | 478.6  mg/100ml | DP-AA-134 | Pass (95.7%) |
|  |  | Sulfamethoxazol |  | 4500  mg/100ml | 4567.5  mg/100ml | DP-AA-116 | Pass (101.5%) |
|  | S9 | Sulfamethoxypyri dazol sodium | Liquid, yellow, bottle 100ml | 20  g/100ml | 18.63  g/100ml | DP-AA-213 | Pass (93.13%) |
|  |  | Trimethoprim |  | 4  g/100ml | 4.36  g/100ml | DP-AA-131 | Pass (109%) |
|  | S10 | Chlortetracyline HCl | Powder, yellow. Pack 10g | 500  mg/10g | 452.3  mg/10g | DP-AA-28 | Pass (90.5%) |
|  |  | Tiamuline HF |  | 250mg/10g | 245.5  mg/10g | DP-AA-125 | Pass (98.2%) |
|  | S11 | Doxycycline hyclate | Powder, yellow. Pack 100g | 50  g/100g | 47.69  g/100g | DP-AA-48 | Pass (95.4%) |
|  | S12 | Oxytetracycline | Powder, yellow. Pack 50g | 6g/100g | 6.01g/100g | DP-AA-91 | Pass (101.2%) |
|  | S13 | Amoxyclin trihydrate | Powder, white. Pack 100g | 10000  mg/100g | 10966  mg/100g | DP-AA-07 | Pass (109.7%) |
|  | S14 | Ampicilline Trihydrat | Powder, white. Pack 50g | 1250  mg/50g | 1224.6 mg/50g | DP-AA-05 | Pass (97.97%) |
|  |  | Colistin Sulfate |  | 3750000  UI/50g | 3606138.3  UI/50g | DP-AA-20 | Pass (96.2%) |
|  | S15 | Ampicillin | Powder, white. Pack 100g | 87.5g/100g | 15.87  g/100g | DP-AA-05 | Fail (18.16%) |
|  |  | Sulfadimethoxin |  | 12.5g/100g | 11.52  g/100g | DP-AA-113 | Pass (92.2%) |
|  | S16 | Sulfamethoxazole | Powder, white. Pack 20g | 2g/20g | 1.93g/20g | DP-AA-116 | Pass (96.6%) |
|  |  | Trimethoprim |  | 400  mg/20g | 373.9  mg/20g | DP-AA-131 | Pass (93.5%) |
|  | S17 | Sulfachloropyrazine Sodium | Powder, white. Pack 10g | 30g/100g | 9.54g/100g | DP-AA-111 | Fail (31.8%) |
|  | S18 | Diaveridin HCl | Powder, white. Pack 20g | 1g/20g | 0.993g/20g | DP-AA-43 | Pass (99.34%) |
|  |  | Sulfaquinoxaline Sodium |  | 3.74g/20g | 3.51g/20g | DP-AA-117 | Pass (93.8%) |
|  | S19 | Oxytetracyclin HCl | Powder, yellow. Pack 10g | 1.8g/10g | 1.72g/10g | DP-AA-91 | Pass (95.4%) |
|  | S20 | Sulfaguanidine | Powder, white. Pack 10g | 1,8g/10g | 1.824g/10g | DP-AA-115 | Pass (101.4%) |
|  |  | Sodium salicylate |  | 1.55g/10g | 1.46g/10g | DP-AA-03 | Pass (94.3%) |
|  | S21 | Erythromycin | Powder, white. Pack 50g | 488.9  mg/100g | 450.6  mg/100g | DP-AA-52 | Pass (92.2%) |
|  |  | Ampicillin |  | 1268 mg/100g | 1153.9  mg/100g | DP-AA-05 | Pass (91%) |
|  | S22 | Colistin sulfate | Powder, white. Pack 50g | 28800000  UI/50g | 26497398  UI/50g | DP-AA-20 | Pass (92%) |
|  |  | Trimethoprim |  | 1068  mg/50g | 1166.7  mg/50g | DP-AA-131 | Pass (109.3%) |
|  | S23 | Doxycycline hyclate | Powder, yellow. Pack 100g | 10g/100g | 9.14g/100g | DP-AA-48 | Pass (91.4%) |
|  |  | Tylosin tartrate |  | 5g/100g | 4.69g/100g | DP-AA-134 | Pass (93.8%) |
|  | S24 | Doxycycline hyclate | Powder, yellow. Pack 100g | 5g/100g | 4.83g/100g | DP-AA-48 | Pass (96.7%) |
|  |  | Tylosin tartrate |  | 5g/100g | 5.11g/100g | DP-AA-134 | Pass (102.3%) |
|  | S25 | Amoxicillin | Powder, pink. Pack 100g | 6.53g/100g | 6.01g/100g | DP-AA-07 | Pass (92.0%) |
|  |  | Tylosin |  | 4.15g/100g | 3.95g/100g | DP-AA-134 | Pass (95.27%) |
|  | S26 | Sulfamethoxazole | Powder, white. Pack 100g | 10g/100g | 10.096  g/100g | DP-AA-116 | Pass (101.0%) |
|  |  | Trimethoprim |  | 2g/100g | 1.91g/100g | DP-AA-131 | Pass (95.3%) |
|  | S27 | Ampicillin tryhydrate | Powder, white. Pack 100g | 5g/100g | 5.36g/100g | DP-AA-05 | Pass (107.1%) |
|  |  | Colistin Sulfate |  | 20000000  UI/100g | 21909485  UI/100g | DP-AA-20 | Pass (109.6%) |
|  | S28 | Oxytracyclin HCl | Liquid, yellow. Bottle 20ml | 20000  mg/100ml | 6373  mg/100ml | DP-AA-91 | Fail (31.87%) |
|  | S29 | Amoxicillin trihydrate | Suspension, white. Bottle 20ml | 3000  mg/20ml | 3001.9  mg/20ml | DP-AA-07 | Pass (100.1%) |
|  | S30 | Thiamphenicol | Liquid, yellow. Bottle 20ml | 5000  mg/100ml | 1851.7  mg/100ml | DP-AA-123 | Fail (37.03%) |
|  |  | Tylosin tartrate |  | 1000  mg/100ml | 1036.5  mg/100ml | DP-AA-134 | Pass (103.6%) |
|  | S31 | Amoxycilline | Suspension, white. Bottle 100ml | 15g/100ml | 13.71  g/100ml | DP-AA-07 | Pass (91.4%) |
|  | S32 | Ampicillin | Powder, white. Bottle 1g | 1000  mg/1000mg | 990.5  mg/1000mg | DP-AA-05 | Pass (99.05%) |
|  | S33 | Thiamphenicol | Liquid, yellow. Bottle 100ml | 10g/100ml | 10.1  g/100ml | DP-AA-123 | Pass (101%) |
|  |  | Oxytetracyclin HCl |  | 5g/100ml | 4.79  g/100ml | DP-AA-91 | Pass (95.87%) |
|  | S34 | Sulfadimidine | Liquid, brown yellow. Bottle 100ml | 200  mg/1ml | 203.2  mg/1ml | DP-AA-114 | Pass (101.6%) |
|  |  | Trimethoprim |  | 40mg/1ml | 41.68  mg/1ml | DP-AA-131 | Pass (104.2%) |
|  | S35 | Tylosin | Liquid, transparent. Bottle 100ml | 500  mg/100ml | 474.9  mg/100ml | DP-AA-134 | Pass (94.98%) |
|  |  | Sulfamethoxazol |  | 4500  mg/100ml | 4535.5  mg/100ml | DP-AA-116 | Pass (100.8%) |
|  | S36 | Florfenicol | Liquid, yellow. Bottle 100ml | 12000  mg/100ml | 10837.4  mg/100ml | DP-AA-58 | Pass (90.3%) |
|  |  | Doxycycline hyclate |  | 8000  mg/100ml | 7260.5  mg/100ml | DP-AA-48 | Pass (90.8%) |
|  | S37 | Oxytetracycline HCl | Liquid, brown yellow. Bottle 100ml | 5000  mg/100ml | 471.3  mg/100ml | DP-AA-91 | Fail (9.45%) |
|  | S38 | Sulfadimidine | Liquid, egg yellow. Bottle 100ml | 200  mg/1ml | 203.4 mg/1ml  mg/1ml | DP-AA-114 | Pass (101.7%) |
|  |  | Trimethoprim |  | 40  mg/1ml | 41.67  mg/ml | DP-AA-131 | Pass (104.2%) |
|  | S39 | Amoxicillin | Suspension, white. Bottle 100ml | 15000  mg/100ml | 13676.5  mg/100ml | DP-AA-07 | Pass (91.2%) |
|  | S40 | Sulfamethoxazol | Liquid, yellow. Bottle 20ml | 10g/100ml | 10.18  g/100ml | DP-AA-116 | Pass (101.8%) |
|  |  | Trimethoprim |  | 2g/100ml | 1.97  g/100ml | DP-AA-131 | Pass (98.68%) |
|  | S41 | Lincomycin HCl | Liquid, yellow. Bottle 100ml | 80mg/1ml | 83.15  mg/1ml | DP-AA-70 | Pass (103.9%) |
|  |  | Sulfamethoxazole |  | 80mg/1ml | 81.12  mg/1ml | DP-AA-116 | Pass (101.4%) |
|  | S42 | Amoxycillin | Suspension, white. Bottle 100ml | 150mg/1ml | 139.4  mg/1ml | DP-AA-07 | Pass (92.94%) |
|  | S43 | Oxytetracycline HCl | Liquid, brown yellow. Bottle 100ml | 5g/100ml | 5.2  g/100ml | DP-AA-91 | Pass (103.9%) |
|  |  | Thiamphenicol |  | 10g/100ml | 9.44  g/100ml | DP-AA-114 | Pass (94.4%) |
|  | S44 | Sulfamethoxazole | Liquid, yellow. Bottle 100ml | 20g/100ml | 21.13  g/100ml | DP-AA-116 | Pass (105.7%) |
|  |  | Trimethoprim |  | 4g/100ml | 4.07  g/100ml | DP-AA-131 | Pass (101.6%) |
|  | S45 | Amoxicilline | Suspension, white. Bottle 100ml | 15g/100ml | 15.79  g/100ml | DP-AA-07 | Pass (105.3%) |
|  | S46 | Sulfadimethoxine | Powder, white. Pack 100g | 10000  mg/100g | 10046.1  mg/100g | DP-AA-113 | Pass (100.5%) |
|  | S47 | Doxycycline | Powder, yellow. Pack 100g | 14g/100g | 13.7g/100g | DP-AA-48 | Pass (97.8%) |
|  |  | Ampiciline |  | 20g/100g | 1.69g/100g | DP-AA-05 | Fail (8.45%) |
|  | S48 | Ampicilline Trihydrat | Powder, white. Pack 100g | 50000  mg/1kg | 48868.7  mg/1kg | DP-AA-05 | Pass (97.7%) |
|  |  | Colistin Sulfate |  | 125000000UI/1kg | 112664251 UI/1kg | DP-AA-20 | Pass (90.13%) |
|  | S49 | Colistin | Powder, white. Pack 100g | 50000000  UI/1kg | 46553301  UI/1kg | DP-AA-20 | Pass (93.1%) |
|  |  | Sulfaguanidine |  | 200g/1kg | 201.65  g/1kg | DP-AA-115 | Pass (100.8%) |
|  | S50 | Amoxicillin trihydrate | Powder, white. Pack 100g | 10g/100g | 9.54g/100g | DP-AA-07 | Pass (95.43%) |
|  |  | Colistin Sulfate |  | 40000000 UI/100g | 39068655 UI/100g | DP-AA-20 | Pass (97.67%) |
|  | S51 | Streptomycin | Powder, white. Pack 50g | 35mg/1g | 35.5mg/1g | DP-AA-118 | Pass (101.5%) |
|  |  | Sulfamethoxazone |  | 45  mg/1g | 40.91  mg/1g | DP-AA-116 | Pass (90.9%) |
|  | S52 | Spiramycin | Powder, yellow. Pack 100g | 20000000 UI/1000g | 21564808UI/1000g | DP-AA-106 | Pass (107.8%) |
|  |  | Oxytetracycline |  | 50  g/1000g | 45.1  g/1000g | DP-AA-91 | Pass (90.2%) |
|  | S53 | Amoxicillin Trihydrate | Powder, white. Pack 100g | 5g/100g | 5.49g/100g | DP-AA-07 | Pass (109.7%) |
|  |  | Colistin Sulfate |  | 40000000  UI/100g | 38379484  UI/100g | DP-AA-20 | Pass (95.95%) |
|  | S54 | Sulfamethoxazole | Powder, yellow. Pack 100 g | 20  g/100g | 19.58  g/100g | DP-AA-116 | Pass (97.9%) |
|  |  | Oxytetracyclin HCl |  | 10g/100g | 9.05g/100g | DP-AA-91 | Pass (90.5%) |
|  | S55 | Doxycycline | Powder, yellow. Pack 100g | 14  g/100g | 13.53  g/100g | DP-AA-48 | Pass (96.6%) |
|  |  | Ampiciline |  | 20g/100g | 2.15g/100g | DP-AA-07 | Fail (10.75%) |
|  | S56 | Doxycycline | Powder, yellow. Pack 50g | 5g/50g | 4.71g/50g | DP-AA-48 | Pass (94.22%) |
|  | S57 | Ampicillin Trihydrate | Powder, white. Pack 50g | 50  g/1000g | 45.86  g/1000g | DP-AA-05 | Pass (91.72%) |
|  |  | Colistine Sulfate |  | 110000000UI/1000g | 119152225  UI/1000g | DP-AA-20 | Pass (108.3%) |
|  | S58 | Ampicilline trihydrate | Powder, white. Pack 50g | 25mg/g | 22.73mg/g | DP-AA-05 | Pass (90.9%) |
|  |  | Colistin Sulfate |  | 50000UI/g | 49610UI/g | DP-AA-20 | Pass (99.2%) |
|  | S59 | Oxytetracycline HCl | Powder, yellow. Pack 1kg | 500000  mg/1kg | 452694.3  mg/1kg | DP-AA-91 | Pass (90,54%) |
|  | S60 | Amoxycyclin Trihydrate | Powder, white. Pack 50g | 2.5g/50g | 2.51g/50g | DP-AA-07 | Pass (100.4%) |
|  | S61 | Trimethoprim | Powder, white. Pack 50g | 30mg/g | 29.87mg/g | DP-AA-131 | Pass (99.6%) |
|  |  | Colistin Sulfate |  | 500000  UI/g | 465831 UI/g | DP-AA-20 | Pass (93.2%) |
|  | S62 | Sulfadimidine | Powder, rose white. Pack 100g | 21.3g/100g | 19.5g/100g | DP-AA-114 | Pass (91.6%) |
|  |  | Diaveridine |  | 2.6g/100g | 2.36g/100g | DP-AA-43 | Pass (90.8%) |
|  | S63 | Sulfachloropyrazine | Powder, barley white. Pack 100g | 300mg/1g | 280.8mg/1g | DP-AA-111 | Pass (93.6%) |
|  | S64 | Sulfadiazine | Liquid, yellow. Bottle 250ml | 400  mg/ml | 399.5  mg/ml | DP-AA-112 | Pass (99.9%) |
|  |  | Trimethoprim |  | 80  mg/ml | 94.3  mg/ml | DP-AA-131 | Pass (105.4%) |
|  | S65 | Oxytetracycline | Liquid, brown yellow. Bottle 100ml | 200  mg/1ml | 205.7  mg/1ml | DP-AA-91 | Pass (102.8%) |
|  | S66 | Ampicillin trihydrate | Suspension, white. Bottle 100ml | 10000  g/100ml | 9772.8 g/100ml | DP-AA-05 | Pass (97.7%) |
|  |  | Colistine Sulfate |  | 25000000  UI/100ml | 23530363 UI/100ml | DP-AA-20 | Pass (94.1%) |
|  | S67 | Thiamphenicol | Liquid, brown yellow. Bottle 100ml | 100  mg/1ml | 107.6  mg/1ml | DP-AA-123 | Pass (107.6%) |
|  |  | Oxytetracycline HCl |  | 50mg/1ml | 54.87mg/ml | DP-AA-91 | Pass (109.7%) |
|  | S68 | Trimethoprim | Liquid, yellow. Bottle 100ml | 4000  mg/100ml | 3995.6  mg/100ml | DP-AA-131 | Pass (99.9%) |
|  |  | Sulfamethoxazole sodium |  | 20000  mg/100ml | 20652.6  mg/100ml | DP-AA-116 | Pass (103.3%) |
|  | S69 | Sulfamethazine Sodium | Liquid, transparent. Bottle 100ml | 330  mg/1ml | 338.1  mg/ml | DP-AA-114 | Pass (102.4%) |
|  | S70 | Amoxicilline | Suspension, white. Bottle 100ml | 15  g/100ml | 14.13  g/100ml | DP-AA-07 | Pass (94.2%) |
|  | S71 | Oxytetracycline | Liquid, brown yellow. Bottle 100ml | 200  mg/1ml | 185  mg/1ml | DP-AA-91 | Pass (92.5%) |
|  | S72 | Amoxicyclin | Suspension, white. Bottle 100ml | 15  g/100ml | 13.74  g/100ml | DP-AA-07 | Pass (91.6%) |
|  | S73 | Tylosin tatrate | Powder, yellow. Pack 100g | 5g/100g | 5.19g/100g | DP-AA-134 | Pass (103.8%) |
|  |  | Doxycycline hyclate |  | 5g/100g | 4.85g/100g | DP-AA-48 | Pass (96.97%) |
|  | S74 | Amoxycillin | Powder, white. Pack 10g | 500  mg/10g | 456.2  mg/10g | DP-AA-07 | Pass (91.24%) |
|  |  | Colistin sulfate |  | 2000000 IU/10g | 2075161  UI/10g | DP-AA-20 | Pass (103.7%) |
|  | S75 | Oxytetracycline | Powder, yellow. Pack 5g | 100mg/g | 91.2mg/g | DP-AA-91 | Pass (91.2%) |
|  | S76 | Sulfadimethoxin | Powder, white. Pack 20g | 28  g/100g | 25.32  g/100g | DP-AA-113 | Pass (90.4%) |
|  |  | Trimethoprim |  | 6g/100g | 5.49g/100g | DP-AA-131 | Pass (91.5%) |
|  | S77 | Amoxycillin trihydrate | Suspension, white. Bottle 20ml | 15  g/100ml | 15.06  g/100ml | DP-AA-07 | Pass (100.4%) |
|  | S78 | Amoxicillin trihydrate | Powder, white. Pack 50g | 200g/1kg | 209g/1kg | DP-AA-07 | Pass (104.5%) |
|  |  | Colistin sulfate |  | 8 MUI/1kg | 7.22  MUI/kg | DP-AA-20 | Pass (90.3%) |
|  | S79 | Trimethoprim | Liquid, yellow. Bottle 100ml | 4  g/100ml | 4.39 g/100ml | DP-AA-131 | Pass (109.9%) |
|  |  | Sunfamethoxazole |  | 20g/100ml | 19.7  g/100ml | DP-AA-116 | Pass (98.5%) |
|  | S80 | Colistine sulphate | Powder, white. Pack 10g | 500000  UI/1g | 479.427 UI/1g | DP-AA-20 | Pass (95.9%) |
|  |  | Trimethoprim |  | 37.5mg/1g | 36.22mg/1g | DP-AA-131 | Pass (96.6%) |
|  | S801 | Doxycycline | Powder, yellow. Pack 10g | 5000  mg/100g | 4548.9  mg/100g | DP-AA-48 | Pass (90.98%) |
|  | S82 | Ampicilline Trihydrate | Powder, white. Pack 10g | 50000  mg/1000g | 48119.7  mg/1000g | DP-AA-05 | Pass (96.2%) |
|  |  | Colistine sulphate |  | 125000000  UI/1000g | 113228285  UI/1000g | DP-AA-20 | Pass (90.6%) |
|  | S83 | Trimethoprim | Powder, white. Pack 20g | 200mg/10g | 184.4  mg/10g | DP-AA-131 | Pass (92.2%) |
|  |  | Sulfamethoxazole |  | 1g/10g | 0.937g/10g | DP-AA-116 | Pass (93.7%) |
|  | S84 | Trimethoprim | Liquid, transparent. Bottle 100ml | 4g/100ml | 1.22  g/100ml | DP-AA-131 | Fail (30.6%) |
|  |  | Sulfamethoxypyri  dazine |  | 20  g/100ml | 4.58  g/100ml | DP-AA-213 | Fail (22.9%) |
|  | S85 | Oxytetracycline | Liquid, brown yellow. Bottle 100ml | 200mg/ml | 189.2mg/ml | DP-AA-91 | Pass (94.58%) |
|  | S86 | Ampicilline | Powder, white. Pack 1g | 1000  mg/1000mg | 992.5 mg/1000mg | DP-AA-05 | Pass (99.3%) |
|  | S87 | Doxycyclin | Liquid, yellow. Bottle 100ml | 10  g/100ml | 9.436  g/100ml | DP-AA-48 | Pass (94.36%) |
|  |  | Florfenicol |  | 10  g/100ml | 10.47  g/100ml | DP-AA-58 | Pass (104.7%) |
|  | S88 | Oxytetracycline | Liquid, brown yellow. Bottle 100ml | 200mg/1ml | 186.9mg/1ml | DP-AA-91 | Pass (93.5%) |
|  | S89 | Sulfamethoxypyridazine | Liquid, yellow. Bottle 20ml | 30  g/100ml | 30.98  g/100ml | DP-AA-213 | Pass (103.3%) |
|  | S90 | Amoxicillin trihydrate | Suspension, white. Bottle 100ml | 15  g/100ml | 13.95  g/100ml | DP-AA-07 | Pass (92.98%) |
|  | S91 | Amoxicillin trihydrate | Suspension, white. Bottle 100ml | 15000  mg/100ml | 15000  mg/100ml | DP-AA-07 | Pass (100%) |
|  | S92 | Amoxicillin | Powder, yellow. Pack 50g | 4.2g/100g | 4.53g/100g | DP-AA-07 | Pass (107.9%) |
|  |  | Colistin |  | 12500000 UI/100g | 13649749  UI/100g | DP-AA-20 | Pass (109.2%) |
|  | S93 | Doxycycline HCL | Powder, yellow. Pack 100g | 20  g/100g | 20.01  g/100g | DP-AA-48 | Pass (100.06%) |
|  | S94 | Sulphachlozine | Powder, white. Pack 10g | 300mg/1g | 326.7mg/1g | DP-AA-109 | Pass (108.9%) |
|  | S95 | Amoxycillin trihydrate | Suspension, white. Bottle 20ml | 150  mg/1ml | 141.2  mg/1ml | DP-AA-07 | Pass (94.12%) |
|  | S96 | Oxytetracycline | Liquid, brown yellow. Bottle 100ml | 200  mg/100ml | 189.1 mg/100ml | DP-AA-91 | Pass (94.56%) |
|  | S97 | Sulphadimidin sodium | Powder, white. Pack 10g | 2000  mg/10g | 1916.3  mg/10g | DP-AA-114 | Pass (95.8%) |
|  |  | Diaveridin |  | 200  mg/10g | 190.23  mg/10g | DP-AA-43 | Pass (95.1%) |
|  | S98 | Amoxycillin | Powder, white. Pack 50g | 2.5g/50g | 2.3g/50g | DP-AA-07 | Pass (91.9%) |
|  |  | Colistin sunfate |  | 10000000 UI/10g | 10355187 UI/10g | DP-AA-20 | Pass (103.6%) |
|  | S99 | Tylosin tatrate | Powder, yellow. Pack 50g | 2.5g/50g | 2.527g/50g | DP-AA-134 | Pass (101.1%) |
|  |  | Doxycycline hyclate |  | 2.5g/50g | 2.3g/50g | DP-AA-48 | Pass (92.2%) |
|  | S100 | Doxycycline HCl | Liquid, yellow. Bottle 100ml | 10 g/100ml | 9.54  g/100ml | DP-AA-48 | Pass (95.38%) |
|  |  | Flofenicol |  | 10 g/100ml | 10.6  g/100ml | DP-AA-58 | Pass (106.1%) |
|  | S101 | Sulphamethoxypyridazine | Liquid, yellow. Bottle 100 ml | 30g/100ml | 31.06  g/100ml | DP-AA-213 | Pass (103.5%) |
|  | S102 | Amoxixillin | Suspension, white. Bottle 20ml | 150 mg/1ml | 143.5  mg/1ml | DP-AA-07 | Pass (95.7%) |
|  | S103 | Amoxixillin (Trihydrat) | Suspension, white. Bottle 20ml | 15000  mg/100ml | 13538.4  mg/100ml | DP-AA-07 | Pass (90.26%) |
|  | S104 | Trimethoprime | Liquid, yellow. Bottle 20ml | 4 g/100ml | 4.34  g/100ml | DP-AA-131 | Pass (108.8%) |
|  |  | Sulfamethoxazole |  | 20 g/100ml | 19.55  g/100ml | DP-AA-116 | Pass (97.7%) |
|  | S105 | Trimethoprime | Liquid, yellow. Bottle 20ml | 4g/100ml | 4.17  g/100ml | DP-AA-131 | Pass (104.3%) |
|  |  | Sulfamethoxine |  | 18.67  g/100ml | 20.4  g/100ml | DP-AA-113 | Pass (109.3%) |
|  | S106 | Doxycycline HCL | Liquid, yellow. Bottle 20ml | 10  g/100ml | 9.72  g/100ml | DP-AA-48 | Pass (97.2%) |
|  |  | Flofenicol |  | 10  g/100ml | 10.89  g/100ml | DP-AA-58 | Pass (108.9%) |
|  | S107 | Tylosin | Powder, yellow. Pack 10g | 10g/100g | 9.08g/100g | DP-AA-134 | Pass (90.8%) |
|  |  | Doxycycline |  | 10g/100g | 9.77g/100g | DP-AA-48 | Pass (97.7%) |
|  | S108 | Ampiciclin trihydrate | Powder, white. Pack 100g | 2250  mg/100g | 1191.4  mg/100g | DP-AA-05 | Fail (52.95%) |
|  |  | Colistin sunfate |  | 29000000 UI/100g | 26329185  UI/100g | DP-AA-20 | Pass (90.8%) |
|  | S109 | Sulfadimethoxin | Powder, white. Pack 20g | 28 g/1000g | 25.36  g/1000g | DP-AA-113 | Pass (90.6%) |
|  |  | Trimethoprim |  | 6g/1000g | 5.83  g/1000g | DP-AA-131 | Pass (97.1%) |
|  | S110 | Sulfaclozin | Powder, barley white. Pack 100g | 30  g/100g | 39.73  g/100g | DP-AA-109 | Pass (99.1%) |
|  | S111 | Amoxixillin (Trihydrat) | Powder, orange -pink. Pack 10g | 6.53g/100g | 6.06g/100g | DP-AA-07 | Pass (92.9%) |
|  |  | Tylosin (Tartrate) |  | 4.15g/100g | 3.96g/100g | DP-AA-134 | Pass (95.5%) |
|  | S112 | Amoxixillin | Powder, white. Pack 100g | 3000  g/100g | 2710.8  g/100g | DP-AA-07 | Pass (90.36%) |
|  |  | Sulfadimidine sodium |  | 5000  g/100g | 5430.4  g/100g | DP-AA-114 | Pass (108.6%) |
|  | S113 | Sulfadimidine | Powder, white. Pack 100g | 400  mg/100g | 414  mg/100g | DP-AA-114 | Pass (103.5%) |
|  |  | Tylosin tartrate |  | 400  mg/100g | 379.9  mg/100g | DP-AA-134 | Pass (94.97%) |
|  | S114 | Oxytetracycline | Powder, yellow. Pack 100g | 10g/100g | 9.28g/100g | DP-AA-91 | Pass (92.8%) |
|  | S115 | Amoxicillin trihydrate | Powder, white. Pack 100g | 20  g/100g | 18.11  g/100g | DP-AA-07 | Pass (90.57%) |
|  |  | Colistin sunfate |  | 60000000 UI/100g | 54114651  UI/100g | DP-AA-20 | Pass (90.2%) |
|  | S116 | Sulfadiazine sodium | Powder, white. Pack 50g | 100mg/1g | 98.95mg/1g | DP-AA-112 | Pass (98.95%) |
|  |  | Trimethoprim |  | 20mg/1g | 20.46mg/1g | DP-AA-131 | Pass (102.3%) |
|  | S117 | Doxycycline hyclate | Powder, yellow. Pack 100g | 500mg/1g | 463.4mg/1g | DP-AA-48 | Pass (92.7%) |
|  | S118 | Doxycycline hyclate | Powder, yellow. Pack 100g | 50  g/100g | 35.22  g/100g | DP-AA-48 | Fail (70.44%) |
|  |  | Colistin sunfate |  | 5000000 UI/100g | 5320923  UI/100g | DP-AA-20 | Pass (106.4%) |
|  | S119 | Amoxixillin | Suspension, white. Bottle 100ml | 150  mg/1ml | 135.3  mg/1ml | DP-AA-07 | Pass (90.21%) |
|  | S120 | Oxytetracycline | Liquid, brown yellow. Bottle 100ml | 200  mg/1ml | 189.8  mg/1ml | DP-AA-91 | Pass (94.9%) |
|  | S121 | Amoxixillin | Suspension, white. Bottle 100ml | 15  g/100ml | 13.72  g/100ml | DP-AA-07 | Pass (91.48%) |
|  | S122 | Sulfamethoxazole | Liquid, yellow. Bottle 100ml | 200  mg/100ml | 205  mg/100ml | DP-AA-116 | Pass (102.5%) |
|  |  | Trimethoprim |  | 40  mg/100ml | 39.08  mg/100ml | DP-AA-131 | Pass (97.7%) |
|  | S123 | Oxytetracycline | Liquid, yellow. Bottle 100ml | 20  g/100ml | 18,7  g/100ml | DP-AA-91 | Pass (93.7%) |
|  | S124 | Amoxixillin (Trihydate) | Suspension, white. Bottle 20ml | 15  g/100ml | 13.72  g/100ml | DP-AA-07 | Pass (91.5%) |
|  | S125 | Sulfamethoxazole | Liquid, yellow. Bottle 100ml | 20  g/100ml | 18.68  g/100ml | DP-AA-116 | Pass (93.4%) |
|  |  | Trimethoprim |  | 4  g/100ml | 4.19  g/100ml | DP-AA-131 | Pass (104.8%) |
|  | S126 | Oxytetracycline HCL | Liquid, yellow. Bottle 100ml | 10  g/100ml | 6.33  g/100ml | DP-AA-91 | Fail (63.3%) |
|  | S127 | Oxytetracycline HCL | Liquid, brown yellow. Bottle 100ml | 10g/100ml | 6.5g/100ml | DP-AA-91 | Fail (65.0%) |
|  | S128 | Oxytetracycline | Liquid, brown yellow. Bottle 100ml | 200  mg/100ml | 188.1  mg/100ml | DP-AA-91 | Pass (94.04%) |
|  | S129 | Doxycycline | Liquid, yellow. Bottle 100ml | 5  g/100ml | 4.78  g/100ml | DP-AA-48 | Pass (95.65%) |
|  |  | Flofenicol |  | 10  g/100ml | 10.69  g/100ml | DP-AA-58 | Pass (106.9%) |
|  | S130 | Oxytetracycline HCL | Powder, yellow. Pack 100g | 10g/100g | 9.1g/100g | DP-AA-91 | Pass (91%) |
|  |  | Streptomycin sulfate |  | 5g/100g | 5.16g/100g | DP-AA-118 | Pass (103.3%) |
|  | S131 | Doxycycline HCL | Powder, yellow. Pack 100g | 20g/100g | 18.1g/100g | DP-AA-48 | Pass (90.43%) |
|  | S132 | Oxytetracycline HCL | Powder, yellow. Pack 100g | 1.25g/100g | 1.13g/100g | DP-AA-91 | Pass (90.7%) |
|  | S133 | Sulfamethoxazole | Liquid, yellow. Bottle 100ml | 20  g/100ml | 20.9  g/100ml | DP-AA-116 | Pass (104.5%) |
|  |  | Trimethoprim |  | 4  g/100ml | 4.03  g/100ml | DP-AA-131 | Pass (100.7%) |
|  | S134 | Sulfamethoxazole | Liquid, yellow. Bottle 100ml | 200  mg/1ml | 204.1  mg/1ml | DP-AA-116 | Pass (102.1%) |
|  |  | Trimethoprim |  | 40  mg/1ml | 38.9  mg/1ml | DP-AA-131 | Pass (97.3%) |
|  | S135 | Sulfadimidin sodium | Liquid, yellow. Bottle 20ml | 2000  mg/20ml | 2035.9  mg/20ml | DP-AA-114 | Pass (101.8%) |
|  | S136 | Trimethoprim | Powder, white. Pack 100g | 2000  mg/100g | 1821.7  mg/100g | DP-AA-131 | Pass (91.1%) |
|  |  | Sulfamethoxazole |  | 5000  mg/100g | 4980.9  mg/100g | DP-AA-116 | Pass (99.6%) |
|  |  | Sulfadiazine |  | 5000  mg/100g | 5099.6  mg/100g | DP-AA-112 | Pass (102%) |
|  |  | Tylosin tartrate |  | 5000  mg/100g | 5108.7  mg/100g | DP-AA-134 | Pass (102.2%) |
|  | S137 | Sulfadimidin | Powder, white. Pack 100g | 10  g/100g | 10.73  g/100g | DP-AA-114 | Pass (107.3%) |
|  | S138 | Sulfadimidin | Powder, white. Pack 100g | 21.3  g/100g | 22.62  g/100g | DP-AA-114 | Pass (106.2%) |
|  |  | Diaveridin |  | 2.6g/100g | 2.36g/100g | DP-AA-43 | Pass (90.84%) |
|  | S139 | Amoxycillin trihydrate | Powder, white. Pack 100g | 15  g/100g | 14.22  g/100g | DP-AA-07 | Pass (94.8%) |
|  | S140 | Ampicillin trihydrate | Powder, white. Pack 50 g | 1500  mg/50g | 1360  mg/50g | DP-AA-05 | Pass (90.7%) |
|  |  | Colistin sulfate |  | 4000000 UI/50g | 3633563.8  UI/50g | DP-AA-20 | Pass (90.8%) |
|  | S141 | Amoxyclin trihydrate | Powder, white. Pack 100g | 10000  mg/100g | 10898.6  mg/100g | DP-AA-07 | Pass (109%) |
|  | S142 | Amoxyclin (trihydrate) | Suspension, white. Bottle 100ml | 15  g/100ml | 13.68  g/100ml | DP-AA-07 | Pass (91.18%) |
|  | S143 | Amoxyclin (trihydrate) | Suspension, white. Bottle 20ml | 150  mg/1ml | 136.4  mg/1ml | DP-AA-07 | Pass (90.93%) |
|  | S144 | Amoxyclin (trihydrate) | Suspension, white. Bottle 20ml | 15000  mg/100ml | 15272.2  mg/100ml | DP-AA-07 | Pass (101.8%) |
